# Spontaneous Expulsion of Intramural Fibroid Six Weeks after Emergency Caesarean Section

**DOI:** 10.1155/2015/640570

**Published:** 2015-08-26

**Authors:** Balvinder Sagoo, Ka Ying Bonnie Ng, G. Ghaleb, Heather Brown

**Affiliations:** ^1^Department of Obstetrics & Gynaecology, London North West Healthcare NHS Trust, UK; ^2^Department of Obstetrics and Gynaecology, Chelsea and Westminster Hospital, UK; ^3^Department of Obstetrics & Gynaecology, Worthing and Southlands Hospital NHS Trust, UK

## Abstract

We present a thirty-six-year-old woman with a high risk pregnancy, complicated by multiple congenital anomalies, severe hyperemesis, a pulmonary embolus, and a large intramural fibroid. This fibroid grew in size during the pregnancy. At 34 + 5 weeks, there were reduced fetal movements and a pathological CTG. A live infant was delivered by an emergency cesarean section. Five weeks postpartum, she presented with abdominal pain, offensive vaginal discharge, and fevers. She was given antibiotics and ferrous sulphate. An abdominal ultrasound showed an 11 × 12 × 9 cm fibroid with a coarse degenerative appearance. Clinically, she showed signs of sepsis; a CT scan and laparotomy performed under general anesthetic did not find any collections as a source of sepsis. When stable, she was discharged. She re-presented two days later with a large mass (necrotic fibroid) in her vagina. This is the first case of spontaneous expulsion of fibroid six weeks after caesarean section. Presentation of pain and fever after the delivery may be due to red degeneration of the fibroid, caused by diminished blood supply, ischaemia, and necrosis. This case highlights the importance of considering fibroids as a cause for abdominal pain during and after pregnancy, even up to 6 weeks after delivery.

## 1. Introduction

Uterine fibroids or leiomyomas are the commonest benign tumors amongst women, and, by 50 years of age, almost 70% of white women and more than 80% of black women will have one or more fibroids [1]. They are monoclonal tumors of the smooth muscle cells of the uterus, consisting of extracellular matrix, which contains a mix of collagen, fibronectin, and proteoglycan [2, 3]. There is evidence to suggest that growth of the tumor is accelerated by the hormones progesterone and oestrogen [4, 5]; they rarely occur prior to menarche [6] and, after the menopause, they regress [7]. Fibroids cause functional disturbance of the uterus and severe symptoms, including excessive uterine bleeding, anaemia, defective embryo implantation, recurrent miscarriages, prematurity, obstruction of labor, pelvic pain, and urinary incontinence, which manifest in 15–30% of women [1].

The prevalence of uterine fibroids during pregnancy is likely to be underestimated due to the difficulty with the differentiation of fibroids and physiological myometrium thickening [8]. Studies have shown that the majority of fibroids during pregnancy do not significantly change in volume [9]. Pain is the commonest complication of fibroids during pregnancy and is more common in second and third trimesters of pregnancy and in women with large fibroids (greater than 5 cm) [10, 11].

Previous cases have reported spontaneous expulsion of fibroids one day to two weeks postpartum [12, 13] or immediately after delivery of the baby [14]. This is the first reported case in the published literature of spontaneous postpartum expulsion of an intramural fibroid six weeks after an emergency caesarean section.

## 2. Case Study

This was a thirty-six-year-old multiparous woman (two normal deliveries seventeen and eighteen years ago, with a previous partner) under antenatal care in our department. She was categorized as high-risk pregnant due to her baby being diagnosed with multiple congenital anomalies at the routine anomaly scan. Her pregnancy was also complicated with severe hyperemesis and a pulmonary embolus that had been successfully managed medically with low molecular weight heparin (LMWH).

At the routine dating scan (at 11 + 6 weeks), a large intramural fibroid was seen. Due to the multiple congenital fetal anomalies, our patient was followed up with regular growth scans every 4 weeks; on these scans, the fibroid was seen to be growing significantly (from six to nineteen cm in diameter).

At thirty-four weeks and five days, the patient described reduced fetal movements. Assessment in fetal assessment unit (FAU) with a cardiotocograph (CTG) demonstrated a pathological trace (baseline above 160 bpm, accelerations present, and decelerations with no evidence of uterine activity).

Consequently she underwent an uncomplicated category one emergency caesarean section delivering a live male infant who had Apgar scores of 6^1^, 9^5^, and 9^10^, and there was no obvious reason for the pathological CTG. Because of the multiple congenital abnormalities (oesophageal atresia and transoesophageal fistula) as well as severe intrauterine growth restriction, the baby was immediately transferred to a tertiary unit (Great Ormond Street Hospital, London) and the mother, who had an uneventful postoperative recovery, was discharged from the ward one day later to join her baby.

Five weeks postpartum she was referred to hospital by her general practitioner with persistent abdominal cramps and pain with offensive vaginal discharge, which had not eased since her caesarean section. She also had systemic symptoms of infection such as decreased appetite, generalized malaise, and lethargy. Abdominal palpation found a soft abdomen with no guarding, but she was uncomfortable with a 20-week firm, nonmobile mass of the abdomen. An internal vaginal examination and speculum revealed normal vulva, vagina, and cervix but there was an offensive discharge and a microbiology swab was taken.

She was admitted with a working diagnosis of red degeneration of the fibroid. During her stay, she was treated with ferrous sulphate for anaemia and antibiotics for a suspected infection. She had swinging pyrexia that did not settle with intravenous Tazocin, an antibiotic to which the bacteria cultured from the vaginal swab was sensitive; this antibiotic was also recommended by the microbiologist. Abdominal ultrasound scans did not find any evidence of collection or free fluid and the fibroid was measured eleven by twelve by nine cm with a coarse degenerative appearance. She then went on to have a CT scan, which again did not show an obvious collection that could suggest that the bacteria grown on the HVS could be present in the abdomen. Her pain and pyrexia did not settle so after a MDT discussion with the gynaecology team and microbiologists she underwent a vaginal examination and laparotomy six days into admission, which again did not find a source of infection causing the swinging pyrexia nor an intracavitary fibroid. Under the advice of the microbiologist, she was started on meropenem; eventually the pyrexia and pain settled and she was discharged home once she had been apyrexial for 48 hours.

**Figure 1:**
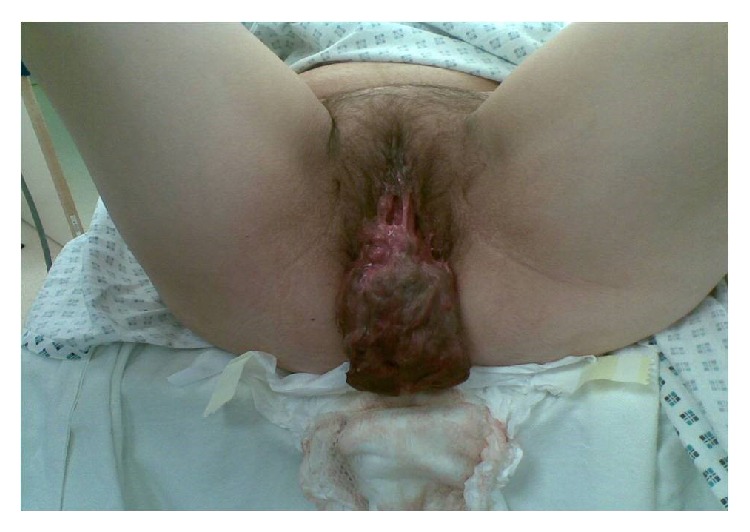
Large necrotic fibroid which was expelled with a long pedicle hanging from the uterine fundus, approximately six weeks after an emergency caesarean section.

Two days after discharge from hospital, she presented complaining of a mass in her vagina. On examination, a large fleshy vaginal mass was protruding from her vagina with no obvious bleeding or discharge; after consenting for an examination under anaesthesia, she was taken to theatre, where the mass was removed. In theatre, a large necrotic fibroid was identified and removed through the vagina by twisting off the long pedicle that was hanging from the uterine fundus (Figure 1, picture with consent from patient).

## 3. Discussion

This is the first reported case of spontaneous expulsion of fibroid six weeks after caesarean section. In our case, we cannot say if the fibroid contributed to the preterm labor or the congenital abnormalities; most likely the latter was the cause. With respect to the fibroid, we feel that there are important learning points from the management of the case after delivery as there are questions about the subsequent interpretation of investigation results and hence the diagnosis. There have been two previous reports of submucous fibroids sloughing in the postnatal phase up to two weeks after delivery of the baby [12, 15] and one case report on spontaneous expulsion of a submucous fibroid soon after delivery of the preterm baby [14].

Fibroids are the most common benign pelvic tumor found in women [1]. In most cases, fibroids are asymptomatic or undetectable clinically during pregnancy. Ultrasound studies show that about 20% of fibroids enlarge during pregnancy and a similar proportion decrease in size [16]. Our patient had a significant increase in the size of her fibroid during pregnancy from 6 cm to 19 cm in diameter at its largest. During the puerperium, the majority of fibroids do not show change; however, about 8% will reduce in volume [17]. There are several complications of fibroids during pregnancy, including preterm labor, preterm prelabor rupture of membranes, spontaneous miscarriage, placenta abruption, or malpresentation [18, 19]. Less commonly, when red degeneration of the fibroid occurs, women present with severe abdominal pain [20]. This occurs because the blood supply can no longer support the growing fibroid resulting in a lack of oxygen and nutrients causing the fibroid to turn red and break down [11]. There is also evidence to support possible “kinking” of the blood vessels as the uterus changes shape or grows, leading to ischaemia and necrosis of the fibroid [3, 21].

Interestingly in this case the patient did not experience any pain prior to or during her pregnancy; she only presented with pain after delivery and the red degeneration is most likely to have occurred in this postpartum phase. In the puerperium period, red degeneration may be more common due to a diminished blood supply to the fibroid after delivery, leading to necrosis.

Prior to delivery, the patient presented with uterine irritability and unprovoked decelerations on CTG. A pregnant uterus complicated with fibroids tends to have decreased oxytocinase activity which can lead to a localized increase in oxytocin levels and preterm contractions [22]. The unprovoked decelerations in our case were most likely due to the fetus being unable to cope with preterm labor due to severe fetal growth restriction and congenital malformations; no other cause for fetal distress was found. Rarely, large submucosal fibroids can compress the uterine cavity and cause fetal deformities. A number of fetal anomalies have been recorded, including dolichocephaly (compression of the fetal skull), torticollis (pathological twisting of the neck), and limb defects [23, 24]. It is unclear whether compression and distortion of the intrauterine cavity in our patient contributed to the congenital anomalies, oesophageal atresia, and transoesophageal fistula. The baby in this case was delivered by an emergency caesarean section, due to a pathological CTG. There are numerous studies showing uterine fibroids as a risk factor for caesarean sections, with approximately a 3.7-fold increase in risk; malpresentation, dysfunctional labor, and placental abruption are the main contributing factors [10, 25, 26].

On repeated scan reports, the fibroid was documented as intramural or subserosal; however, during the caesarean section, it was noted to be submucosal. To our knowledge, there have not been any documented cases of fibroids changing position from intramural to submucosal in the literature to date. The scan report may have been incorrect. Interestingly, during the first few months of pregnancy, there is usually thinning of the uterine wall; in this case, the uterine wall thinned to 3.5 mm, and in the case of uncertainty, it is important for trainees and consultants to be liaise with the consultant radiologist when scans cannot be interpreted reliably. The thinning of the uterine cavity is the only possible explanation for how an intramural fibroid which was completely surrounded by thick uterine muscle could have become submucosal. The red degeneration and expulsion of the fibroid could be explained by involution of the uterus postpartum to regain its prepregnancy size, hence pushing the intramural fibroid towards the line of the least resistance (uterine cavity) and consequently interrupting the blood supply to the fibroid [27].

Our patient developed pyrexia after caesarean section; the most logical reason was infection and, despite administration of appropriate antibiotics, our patient continued to deteriorate clinically. Once the fibroid had been expelled, the patient improved very quickly. Whilst degeneration of a fibroid will remain an unusual cause of postpartum pyrexia and sepsis, it is useful to consider this as a cause. In this case, the patient may have avoided a laparotomy and an extended hospital admission if the team had simply remembered her fibroid and considered that it may have been responsible for the puerperal sepsis. This highlights the importance of thoroughly reviewing patient notes and all the investigations that were done from the beginning of the pregnancy. Nowadays, scans are of high resolution and can be utilized as valuable diagnostic tool in conjunction with a good history from the patient.
